# Rattle Snake’s Poison, and Its Remedies

**Published:** 1871-09

**Authors:** W. T. Grant

**Affiliations:** Ga.


THE GEORGIA

### MEDICAL COMPANION:

A MONTHLY ADVISER.

VOL. I. ATLANTA, GA., SEPTEMBER, 1871. NO. 9

### PART I.

### Original Communications and Special Selections.

### THE RATTLE SNAKE'S POISON, AND ITS REMEDIES.

BY W. T. GRANT, M. D., GA.

The writer has in his possession a copy of an old Magazine printed in London in the year 1766. It is volume 35, complete, containing the monthly numbers from January to December.

It would seem, from the amount of purely medical matter contained in this volume, that there were no medical journals in those days, and that Medical Essayists published wherever they could.

I extract the following article from the September number of this magazine, because it recommends a simple remedy for a very serious difficulty; and with the hope that the Editors will accept it in lieu of such poor original articles as I could write:

"Extract of a letter from Mr. Benjamin Gale, a physician in New England, to John Huxham, M. D., F. R. S., concerning the successful application of salt to wounds made by the biting of Rattle Snakes; dated at Killingworth, in Connecticut, 20th August, 1764, (read June 13th, 1765).

"I have been disappointed in procuring a rattle snake, to make experiments in expelling the poison, particularly the efficacy of sea salt; but have now the satisfaction to acquaint you, that, having desired Mr. Porter, an eminent Surgeon, and a gentleman of worth and probity, to make inquiry, whether the efficacy of sea salt could be properly attested; he, this day, informs me that a person was wounded by that serpent, about the beginning of this month, just above his shoe. The teeth of this serpent, upon examination by the probe, he found to have entered near half an inch. The person bitten immediately made a strong ligature above the wound, and in less than two hours came to Mr. Porter's. The leg and foot below the ligature were much swelled, and the patient grievously affected with a nausea. Mr. Porter made immediately a deep scarification, rubbed it with salt, applied a dossil of lint, moistened over the salt, and placed it over the scarifications, and dismissed his patient, who the next morning returned. The ligature was continued, nevertheless, the tumifaction was greatly abated; the dressing, before applied, was renewed, and the person recovered without any further application. This, perhaps, together with the former instance, may serve to establish the truth of its efficacy."

The "former instance" mentioned in the last sentence above, is related in a foot-note, and is as follows:

"This was a person under the care of Mr. Strong, Surgeon in New England, who, in the year 1761, was bitten by a rattle-snake in the left foot, between the great toe and the next. He immediately perceived a sickness at the stomach, which continued sometime. Scarifications were directly made, by cutting the skin, pulled up by an awl, formed into a hook for the purpose. The first application was fine sea-salt, which was plentifully sprinkled, and rubbed in and about the wound and scarification. These were done in the space of about two minutes after the wound was made. Then a poultice, made of Burdock root pounded, and mixed with a large portion of sea-salt, was applied to the wound, and another of blood root was bound about his leg a little below the knee. In the meantime, the patient took, inwardly, saffron and water, in which was steeped in the bark of white oak, which caused him to vomit. The consequence of the wound was a tumifaction, which was greatest in the foot, but extended to the knee, when it ended. After these applications, nothing remarkable was observed in the wound. They were continued for two days, and the patient perfectly cured. Mr. Strong supposed the salt to be the principal ingredient which effected the cure."

Some years since I was called on to see a negro boy who said he was bitten by a highland moccason. While hunting for it to kill it, he stepped on it, and saw it the same instant. He said he saw it strike and felt the sting. I scarified the place and applied a poultice of common cooking soda, and gave him brandy freely. There were absolutely no results from the bite. No swelling, no pain, no nausea, no anything which could arise from the bite of a poisonous snake. But the boy killed the snake, and I saw it. Whether the treatment, which was applied in five minutes, was as efficacious as would appear from this statement, I cannot say. I mention this case to call attention to the fact that soda is also the basil element in salt, and this makes the treatment of my case and the others quoted above, clearly akin.

In lower Georgia it is believed that a person bitten by a rattlesnake, can cure himself by *eating tobacco*, and I have had quite a number of cases related to me of its efficacy, with time, place and names all given. Some of the cases were related by eye-witnesses. It is stated that the tobacco does not sicken the stomach, and as soon as it does the person is safe.

I know several plants which also possses reputed curative virtues in such cases. And if the evidences of exprofessional persons can be received, there is abundant evidences furnished me to have faith in some of them, at least. I have thought of writing one or more articles upon the subject of "Domestic Remedies," and may at some time do so. I can only give the names of some of those plants at present. The following are some of them: *Platanthera Stricta*, *Amsonia Ciliata*, and *Raptisia Perfoliata.* One of the Euphorbias, which is very abundant in Lower Georgia, also possesses this power of neutralizing the poison of the Rattlesnake. Its specific name I cannot now give, as I never examined it closely, although I know the plant very well.

I have wished to test these reputed virtues by experiment, but up to this time, I have never had an opportunity to do it. I do not think that these plants grow above the lower edge of Middle Georgia—I have never seen any of them anywhere except below the middle portion of the State. There are other experiments I would like to make with our indigenous remedies, and if it shall ever be in my power to execute this wish, I shall certainly let the results be known. While I repudiate the charge of being a "Botanic" or "Root-Doctor," I am not willing that that class of medical practitioners shall steal our thunder and parade themselves before the world as the only ones who use Botanic medicines. I think it is susceptable of proof that the "Botanics," properly so-called, have never yet discovered, or introduced into medicine a single first-class medicine. Their best remedies were in use long before their founder was born. And as for their doctrine of not using any medicines not of vegetable origin (which, however, is not always their practice,) I cannot but think it a contracted view to take of the subject. The true doctrine—the doctrine whose simple statement carries conviction with it—is to use anything under the sun that will relieve disease, without regard to its source. And I think this is the doctrine of every true physician.

